# Potential role of the Eph/ephrin system in colorectal cancer: emerging druggable molecular targets

**DOI:** 10.3389/fonc.2024.1275330

**Published:** 2024-04-02

**Authors:** João Figueira Scarini, Moisés Willian Aparecido Gonçalves, Reydson Alcides de Lima-Souza, Luccas Lavareze, Talita de Carvalho Kimura, Ching-Chu Yang, Albina Altemani, Fernanda Viviane Mariano, Heloisa Prado Soares, Gary Chris Fillmore, Erika Said Abu Egal

**Affiliations:** ^1^ Department of Pathology, Faculty of Medical Sciences, University of Campinas (UNICAMP), Campinas, São Paulo, Brazil; ^2^ Department of Oral Diagnosis, Piracicaba Dental School, University of Campinas (UNICAMP), Piracicaba, São Paulo, Brazil; ^3^ Department of Pathology, School of Medicine, University of Utah (UU), Salt Lake City, UT, United States; ^4^ Division of Oncology, Department of Internal Medicine, Huntsman Cancer Institute, University of Utah (UU), Salt Lake City, UT, United States; ^5^ Biorepository and Molecular Pathology, Huntsman Cancer Institute, University of Utah (UU), Salt Lake City, UT, United States

**Keywords:** colorectal cancer (CRC), Eph/ephrin system, pathogenesis, tumor progression, neoangiogenesis, malignant transformation

## Abstract

The Eph/ephrin system regulates many developmental processes and adult tissue homeostasis. In colorectal cancer (CRC), it is involved in different processes including tumorigenesis, tumor angiogenesis, metastasis development, and cancer stem cell regeneration. However, conflicting data regarding Eph receptors in CRC, especially in its putative role as an oncogene or a suppressor gene, make the precise role of Eph-ephrin interaction confusing in CRC development. In this review, we provide an overview of the literature and highlight evidence that collaborates with these ambiguous roles of the Eph/ephrin system in CRC, as well as the molecular findings that represent promising therapeutic targets.

## 1 Introduction

Colorectal cancer (CRC) is a group of malignant tumors that originate in the colon, rectum, and anus. Currently, CRC is the third most common malignant neoplasm in men sex (1,065,960 cases - 23.4% of the total) and is considered the second leading cause of cancer death worldwide (9% of all cancers) (1). In 2023, 153,020 new cases of CRC were diagnosed in the United States, with an estimated mortality rate of 52,550 deaths throughout the year (2).

**Figure 1:**
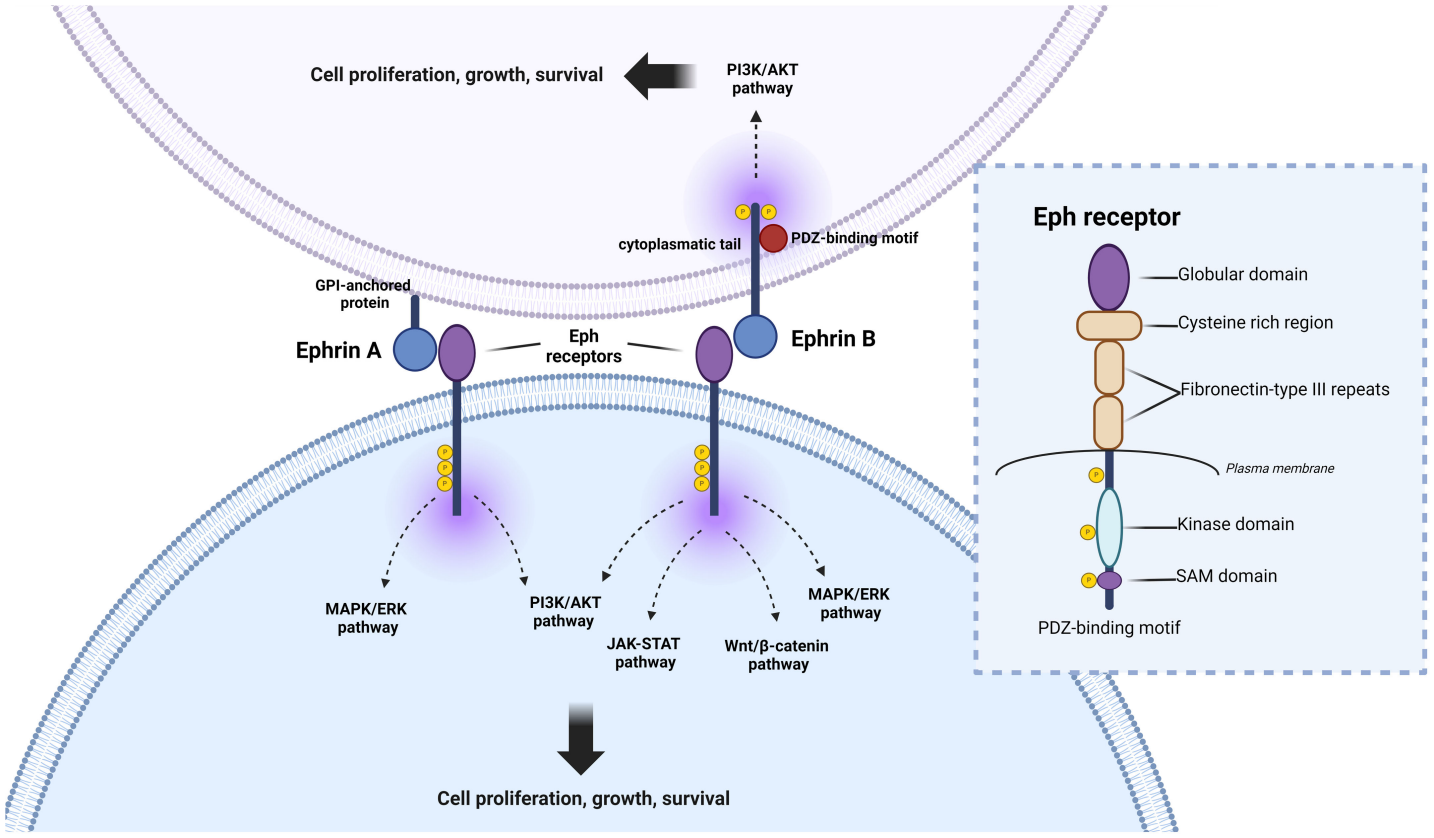
Eph signaling, and pathways involved in colorectal cancer (CRC) progression. Eph receptors participate in intricate crosstalk with various oncogenic pathways, crucially shaping the initiation and perpetuation of CRC. Upon ligand binding, Eph receptors can initiate “forward signaling,” influencing cell repulsion, migration, and invasion—key processes in CRC metastasis. Additionally, EphB receptors can engage in “reverse signaling,” transmitting signals to neighboring or ligand-expressing cells, thereby impacting cell adhesion, migration, and tissue architecture, further influencing CRC progression. Eph receptors are involved in the activation of the MAPK pathway, a signaling cascade pivotal for regulating cell growth, proliferation, and survival. This interaction often promotes tumor growth and metastasis in CRC. Furthermore, Eph receptors modulate the WNT/β-catenin pathway, a central player in CRC pathogenesis. Dysregulation of WNT/β-catenin signaling, along with the JAK-STAT pathway, can fuel aberrant cell proliferation and contribute to CRC formation. Moreover, Eph receptors activate the phosphoinositide 3-kinase (PI3K) signaling pathway, closely linked to cell survival, growth, and angiogenesis. Dysregulation of this pathway may fuel CRC development and confer resistance to therapy. Understanding the intricate interplay between Eph signaling and these pathways is paramount in deciphering the complexities of CRC progression and may offer promising targets for therapeutic intervention. Detailed structural analysis of Eph receptors reveals three distinct regions: (1) Extracellular region, comprising a ligand-binding domain, cysteine-rich domain, and two fibronectin-III like domains; (2) Transmembrane domain; and (3) Intracellular region, consisting of a kinase domain, sterile alpha motif (SAM), and a PDZ binding motif. Both ephrinA (anchored by GPI) and ephrinB (transmembrane) ligands engage with the ligand-binding domain of the Eph receptor.

The Eph (erythropoietin-producing hepatocellular carcinoma) receptor family is the largest subgroup within the tyrosine protein kinase receptor family, potentially influencing physiology and disease through their interaction with ephrin and generation of bidirectional signals at cell-cell contact sites (3) (**Figure 1**). In the normal gastrointestinal tract, the expression gradient of EphA and EphB receptors, as well as ephrins, appears to be distinct along with the crypts and villi (4). EphA1, EphA4, EphA7, EphB1, EphB2, EphB3, EphB4, and EphB6 are present at the base of the crypt, whereas EphA2, EphA5, ephrin A1, and ephrin B2 are expressed at the top of the colon (4). A full understanding of the expression patterns of EphA3, EphA6 and EphA8 in this context may require further specific and dedicated studies. EphB signaling coordinates cell migration and proliferation of intestinal stem cells, which controls cell positioning and regulates the sorting of intestinal cells in the mature epithelium (5–7).

In the last years, Eph system were correlated with the cancer development, cancer progression and drug resistance (8). In colorectal adenomas, the interaction of Eph system limit the tumor expansion and suppress early-stage tumor progression (9). Indeed, previous studies have already demonstrated a tumor suppressor role for EphB and ephrin-B (10–13). In CRC, in contrast to small bowel cancer, EphB-positive tumor cells can reach the surface of the epithelium where normal cells express high levels of ephrin-B (9). The compact anatomy of the crypts in the colon and the small amount of intervillous space cause EphB-positive tumor cells and normal ephrin-B-positive colonocytes to constantly interact (9).

**Figure 2:**
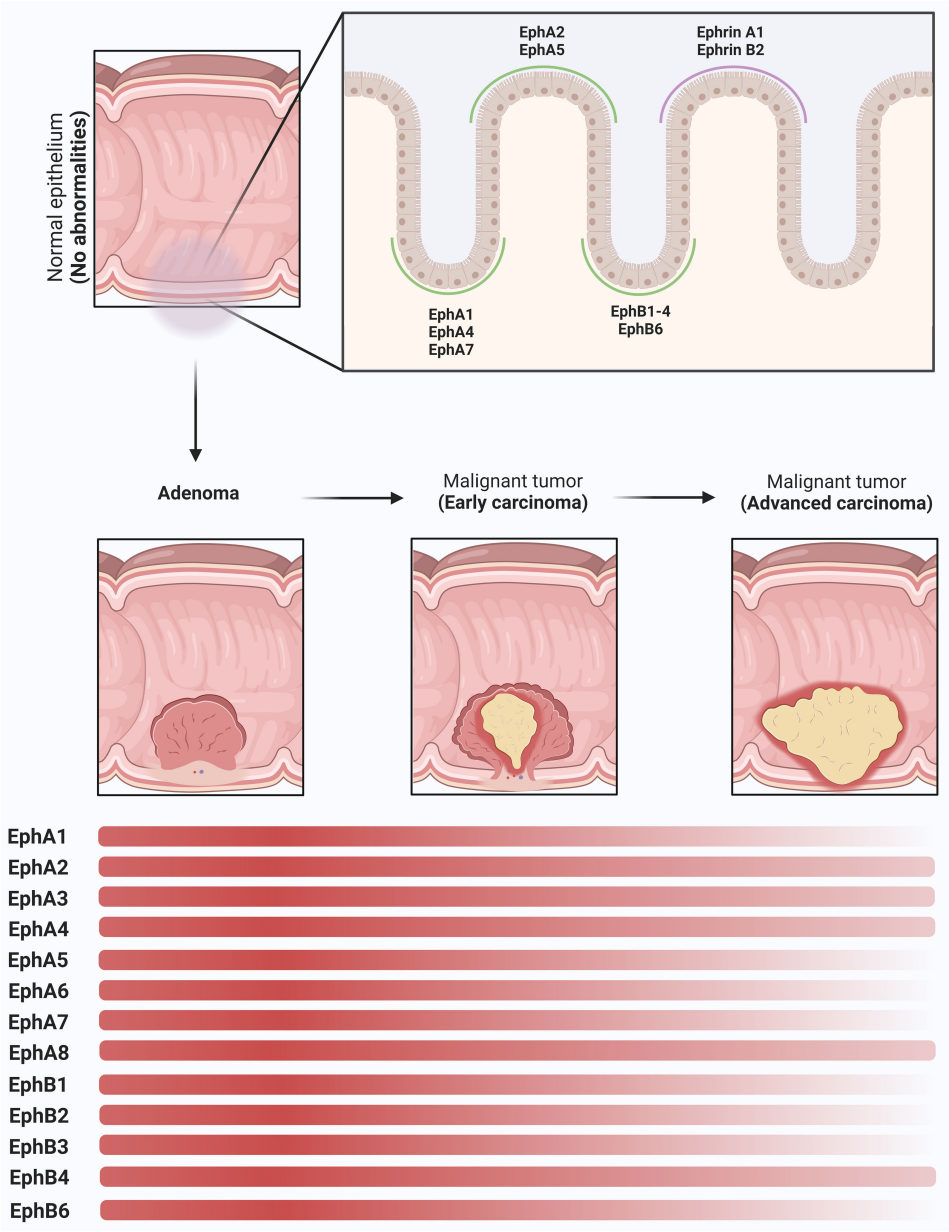
Modulation of Eph receptors and Ephrins in colorectal (CRC) carcinogenesis. In the normal colon tissue, Eph receptors and ephrins play a pivotal role in the regulation of cellular migration, adhesion dynamics, and the maintenance of intestinal mucosal integrity. A distinct spatial gradient in the expression patterns of EphA and EphB receptor subtypes, in addition to ephrins, along the crypt-villus axis is observed. In the basal region of the crypt, EphA1, EphA4, EphA7, EphB1, EphB2, EphB3, EphB4, and EphB6 receptors show increased expression. EphA2, EphA5, ephrin-A1, and ephrin-B2 are prominently expressed in the apical domain of the colonic epithelium. As the adenoma progresses, a remarkable upregulation of these molecules is observed, resulting in cellular proliferation. In the early stages of CRC, a dysregulation in the expression profiles of selected Eph receptors, particularly those endowed with tumor-suppressive functions, becomes apparent, characterized by their decreased expression levels (EphA1, EphA5, EphA6, EphA7, EphB1, EphB2, EphB3, EphB6). EphA2, EphA3, EphA4, EphA8 and EphB4 remain in a state of overexpression and represent the most highly expressed receptor subtypes in advanced stages of CRC. This persistent overexpression promotes the maintenance of tumor invasion and facilitates the formation of metastatic lesions. The depicted graph highlights the expression dynamics of these molecules along the adenoma-to-CRC sequence, revealing a dynamic shift in the expression gradient represented by the transition of the red bars. These intricate changes in Eph and ephrin expression during these successive stages of CRC carcinogenesis underscore the nuanced and multifaceted roles that this protein family plays in the CRC.

In general, at least in the early stages of carcinogenesis, CRC typically shows increased expression of EphA2, EphA3, EphA8 and EphB4 (14–18), whereas EphA6, EphA7, and EphB1 show decreased expression (19–21). On the other hand, the expression levels of EphA1, EphB2, and EphB3 vary between studies, and this has implications for disease progression (18, 20–22) (**Figure 2**). Given the conflicting data regarding Eph receptors in CRC, here we highlight evidence that supports these ambiguous roles of the Eph/ephrin system in these tumors.

## 2 Eph/ephrin axis overview

The Eph family comprises two major groups, EphA (EphA1 to A8 and EphA10) and EphB (EphB1 to EphB6), which are distinguished by sequence homology (23, 24). Structurally, Eph receptors have three distinct regions: the extracellular region, the transmembrane domain, and the intracellular region. The extracellular region contains a ligand-binding domain, a cysteine-rich region, and two fibronectin III repeats, while the transmembrane domain forms a short helix that bridges the extracellular and intracellular domains across the membrane. The intracellular region contains a tyrosine kinase domain, a sterile alpha motif (SAM), and a PDZ-binding motif (25, 26) (**Figure 1**).

Eph receptors bind to ephrins, which are divided into ephrin-A (ephrin-A1 to A6) and ephrin-B (ephrin-B1 to B3) classes (27). Ephrin-A molecules are anchored to the membrane by glycosylphosphatidylinositol (GPI), whereas ephrin-B molecules are characterized as transmembrane proteins with a short cytoplasmic domain and a conserved cytoplasmic tail. In addition, ephrin-A has an affinity for type A ephrin receptors (EphA1 to EphA8 and EphA10), whereas ephrin-B proteins exhibit specific binding to type B ephrin receptors (EphB1 to EphB6) (28).

The intricate cellular communication system revealed by interactions between Ephs and ephrin ligands of the same subclass is promiscuous, but cross-linking between subclasses is less frequently observed. Notable exceptions include EphA3 interactions with ephrin-B2, EphB2 with ephrin-A5, and EphA4 with all 9 ephrins, suggesting a significant degree of cellular plasticity and potential modulation of specificities by genetic alterations. For example, oncogenic mutations in EphA3 have been observed to reduce binding energies with ephrin ligands (A1, A2, A5, and B2), independent of the direct Eph-ephrin interface (29). This aspect adds another layer of complexity to the understanding of these molecules, especially in the context of cancer research. In addition, it is noteworthy that the Eph/ephrin system manifests bidirectional signaling, where the interaction between Eph receptors on one cell and ephrins on another cell triggers signaling in both the receptor-expressing cell and the ephrin-expressing cell (30, 31).

## 3 EphA receptors

### 3.1 EphA1

Among the Eph receptors, EphA1 has received comparatively less attention in human cancer research (32). Asadian et al. (2022) showed no evidence of an association between *EPHA1* polymorphism and CRC risk (33). Liu and colleagues (2022) identified six subtypes of CRC through a network of genetic interaction perturbations. The GINS3 subtype, characterized by KRAS inactivation in 13% to 20% of cases, showed EGFR receptor and ephrin activation, chromosomal instability, and both immunotherapeutic resistance and sensitivity to cetuximab and bevacizumab (34). Single nucleotide polymorphism (SNP) analysis revealed allelic differences between CRC patients and controls. Furthermore, the correlation between *EFNA1* SNP genotyping and tumor characteristics showed that polymorphisms in the *EFNA1* gene may influence the likelihood of tumorigenesis and the risk of disease progression (35).

Recent genetic studies have shown a direct correlation between the loss of *EPHA1* expression and the progression of CRC to a more invasive phenotype (10, 36) (**Table 1**). Low expression of EphA1 protein in CRC has been shown to correlate with invasion, metastasis, and poor overall survival (20). Indeed, the blockade of *EPHA1* by CRISPR/CAS9 can promote the adhesion and motility of CRC cells through the involvement of ERK and JNK signaling pathways (66) (**Figure 1**). Ephrin-A1 is the high-affinity ligand for EphA1 and EphA2 (10). Reduced expression of *EFNA1* in CRC cell lines inhibited invasion ability (67, 68). In mouse models, increased ephrin-A1 expression accelerated malignant progression from adenoma to invasive CRC (37). A study by Ieguchi et al. demonstrated that mice with CRC had increased levels of ephrin-A1 in their serum and urine, and that tumor-derived ephrin-A1 could be a valuable biomarker for predicting pulmonary metastasis in primary tumors expressing high levels of ephrin-A1. Notably, the study also made a significant contribution by identifying for the first time the specific cleavage site of ephrin-A1 by the protease ADAM12 (69). Furthermore, it has been shown that epigenetic silencing of *EPHA1* expression in CRC correlates with poor survival (36)(**Table 2**).

**Table 1:** Roles of various Eph receptors in CRC and their correlation based on current research.

Eph receptors	Role in CRC	Association with CRC	Reference
EphA1	Suppressor role	Decreased of *EPHA1* expression has been shown to correlate with invasion, metastasis, and poor overall survival in CRC	(20, 36, 37)
EphA2	Oncogenic role	Although it seems to act in the early stages of carcinogenesis, it also plays an important role in advanced disease. EphA2 expression had a statistically significant relationship with liver metastasis, lymphatic vessel invasion, and clinical staging	(16, 38, 39)
EphA3	Oncogenic role	Although it seems to act in the early stages of carcinogenesis, it also plays an important role in advanced disease. The loss of *EPHA3* expression was associated with a poor prognosis	(40, 41)
	Suppressor role		
EphA4	Oncogenic role	Although it seems to act in the early stages of carcinogenesis, it also plays an important role in advanced disease. Overexpression of *EPHA4* is related to the promotion of liver metastasis	(42, 43)
	Suppressor role		
EphA5	Suppressor role	Reduced EphA5 expression has been associated with nodal metastasis, advanced TNM stages, and poor prognosis in CRC, indicating that EphA5 may be acting as a tumor suppressor. Indeed, *EFNA5* expression has been correlated with attenuating CRC cell migration and invasion	(44–46)
EphA6	Suppressor role	Expression decreases with disease progression	(47, 48)
EphA7	Suppressor role	Expression decreases with disease progression	(49–51)
EphA8	Oncogenic role	Although it seems to act in the early stages of carcinogenesis, it also plays an important role in advanced disease. Indeed, high expression of *EPHA8* has been recently demonstrated in patients with lymph node metastasis, markedly with worse prognosis	(52)
	Suppressor role		
EphA10	Further research is needed	No clear correlation with disease stage	(53, 54)
EphB1	Suppressor role	Expression decreases as the tumor become more undifferentiated. Furthermore, cancer cells with reduced EphB1 protein expression showed a higher power of tissue invasion	(55)
EphB2	Suppressor role	Negative expression of *EPHB2* signaling in CRC may be a direct pathogenetic mechanism that leads to loss of tissue architecture and gives the tumor advantages in invasion and metastasis	(5, 9)
EphB3	Suppressor role	*EPHB3* controls cell positioning, restricts cell motility, and acts as a tumor suppressor. Recently, *EPHB3* expression was correlated with better clinical outcomes and longer overall survival, suggesting that *EPHB3* is a prognostic indicator in CRC	(22, 56–59)
EphB4	Oncogenic role	Although it seems to act in the early stages of carcinogenesis, it also plays an important role in advanced disease. Increased *EPHB4* gene expression suggests an intrinsic role of EphB4 in the development of a more aggressive tumor phenotype in CRC. On the other hand, opposite findings show that expression of EphB4 is often reduced or lost in colorectal tumors	(15, 60–62)
	Suppressor role		
EphB6	Further research is needed	Expression decreases with disease progression. Genetic analyses showed that when *EPHB6* overexpression accompanies mutations in the APC gene, the tumor acquires significant advantages in cell proliferation, invasion, and metastasis. On the other hand, reduced levels of EphB6 were associated with advanced disease stages and therefore correlated with a poor prognosis	(40, 63–65)

Eph receptors play diverse roles in CRC, either acting as suppressors, inhibiting cancer growth, or as oncogenic contributors, promoting tumor development. Understanding these roles is crucial for developing targeted therapies in CRC treatment. Some Eph receptors, like EphA10 and EphB6, require further research to determine their specific involvement.

CRC, Colorectal cancer.

**Table 2:** Roles of Eph receptors in CRC and their association to epigenetics.

Eph receptors	Association with CRC and epigenetics	Reference
EphA1	The methylation-induced silencing of the *EPHA1* gene has been associated to tumor progression	(3, 36)
EphA3	The hypermethylation of *EPHA3* can lead to a decrease or absence of EphA3 expression and a poor prognosis	(41)
EphA7	The methylation imparts specific biological and histopathological characteristics to its carcinogenesis and differentiation process	(3, 50)
EphB2	The hypermethylation of the promoter is associated with the high frequency of genomic losses and reinforces the suppressive role	(3, 47, 70, 71)
EphB3	The modification of class I and III histone deacetylases and the loss of active chromatin features appear to be important for maintaining the mechanism of epigenetic inhibition, contributing to tumor suppression	(22, 72)
EphB4	*EPHB4* promoter hypermethylation is a common mechanism of EphB4 inactivation, and it may act as a potential suppressor gene	(3, 61)

CRC, Colorectal cancer.

In summary, the loss of EphA1 expression correlates with advanced CRC stages, including invasion and metastasis, usually leading to a poor prognosis. EphA1’s ligand, ephrin-A1, shows similar effects on CRC progression. These findings highlight the importance of EphA1 and ephrin-A1 in CRC pathogenesis and suggest their potential as therapeutic targets or biomarkers for disease prognosis.

### 3.2 EphA2

*EPHA2* gene expression and protein expression have been evaluated in both normal and CRC samples using a variety of methodologies. These studies demonstrated an increase in *EPHA2* gene and protein expression in the tumor tissues examined (73–75) (**Figure 1**). In addition, EphA2 and ephrin-A1 were significantly more highly expressed in early stage (I and II) and smaller CRC. This finding suggests that both EphA2 and ephrin-A1 may play an important role in the early stages of CRC (16) (**Table 1**). In mice carrying a knockout for the *EphA2* gene, the tumors developed were significantly smaller in both the small and large intestine (73). In addition, as in other tumors, EphA2 exhibits angiogenic potential in CRC by contributing to neovascularization (16) or vasculogenic mimicry (76) in the tumor microenvironment (TME) of these tumors. Indeed, in advanced stages of CRC, where the microenvironment is more sophisticated and complex, EphA2 has been associated with tumor progression (38). Furthermore, EphA2 expression has been identified as a poor prognostic marker in stage II/III CRC and is regulated by the MAPK and RalGDS-RalA pathways, both of which are driven by KRAS (77) (**Figure 1**). In summary, mutations in the Ras protein and the activation of EphA2 by VEGF in the Ras-Raf-MAPK pathway are well established contributors to CRC development and treatment resistance (78).

EphA2 has also been correlated with liver metastasis from CRC (79, 80). In an immunohistochemical study of 194 CRC, EphA2 expression had a statistically significant relationship with liver metastasis, lymphovascular invasion, and clinical staging. Furthermore, EphA2 was significantly more expressed in primary lesions with nodal metastasis than in those without metastasis (39).

Most importantly, the high expression of EphA2 in many CRCs may make it a potential target for therapy in this disease (74, 81). In this regard, high EphA2 receptor expression in CRC modulates a mechanism of primary and acquired resistance to cetuximab (82, 83). Cetuximab is a recombinant chimeric monoclonal IgG1 antibody and inhibitor of the epidermal growth factor receptor (EGFR) (84). Almost all patients who initially respond to cetuximab, become refractory. A study by De Robertis and colleagues showed that *EPHA2* gene expression was significantly associated with poor treatment response (85) and combined EphA2 and EGFR overexpression correlated with resistance, independent of the presence of KRAS mutation (85, 86).

In this context, it is noteworthy that patients with KRAS wild-type metastatic CRC harboring NRAS-activating mutations do not benefit from anti-EGFR therapies. Cuyàs and colleagues showed that suppression of oncogenic signaling without EphA2 receptor-ligand binding can restore the efficacy of cetuximab in NRAS-mutant CRC cells (87). Taken together, these studies provide evidence that a specific EPHA2 inhibitor may contribute to cetuximab resistance in human CRC, as recently demonstrated *in vitro* and *in vivo* by Martini and coworkers (82).

With regard to immunotherapy, a tumor antigen was evaluated in liver metastatic mice transfected with EphA2-positive CRC and showed antitumor effects (88). Using pulsed dendritic cells in combination with an EphA2-derived peptide, the same authors demonstrated a high level of immunity against liver metastases of CRC in mice after immunization (89). In addition, EphA2 appears to be a stress antigen and when expressed with AMP-activated protein kinase (AMPK) led to increased CD3 T lymphocyte infiltration in CRC. This mechanism may explain the improved survival of patients with AMPK activation and shows a higher recognition of malignant cells by the CD3 T lymphocyte (90). Recently, researchers have developed inhibitors targeting EphA2 using NVP-BHG712 and triazine-based molecules. These newly synthesized inhibitors have shown encouraging results in the treatment of CRC and are promising for potential therapeutic applications (91). In the field of precision medicine, a proteomic analysis has identified EphA2 as a promising therapeutic target in CRC cells with acquired resistance to cetuximab caused by *KRAS* alterations (92). These findings are significant and warrant attention, as they highlight the potential impact of these discoveries on the advancement of innovative therapeutic strategies for CRC. However, it is crucial to recognize that further validation and exploration in larger patient cohorts and diverse populations are required to gain a full understanding of the clinical implications associated with these findings.

In summary, EphA2 plays a key role in CRC tumor growth and angiogenesis, resulting in poor prognosis and resistance to cetuximab therapy. EphA2 inhibitors have shown potential in the treatment of CRC, either alone or in combination with immunotherapy. However, further research in larger patient populations is needed to fully understand the clinical significance of targeting EphA2 in the treatment of CRC.

### 3.3 EphA3

The role of EphA3 in CRC remains complex and contradictory. Recently, EphA3 has been implicated in the malignant transformation of colorectal epithelial cells *in vitro* and *in vivo*. These results showed that EphA3 may play a role in the early stage of colorectal epithelial cell carcinogenesis (40) (**Table 1**). Andretta and colleagues showed that neither overexpression nor knockout of EphA3 played and important role in tumorigenesis in CRC cell lines (93). In contrast, CRC tissues showed decreased or absent *EPHA3* expression when compared to normal intestinal tissues (41), possibly through epigenetic mechanisms (41, 94) (**Table 2**). Loss of *EPHA3* expression was associated with a poor prognosis (41). Indeed, other authors have shown by sophisticated genomic methods that somatic mutations in the *EPHA3* gene in CRC tissues could inhibit its suppressive function (95–97). On the other hand, the promoting role of EphA3 in CRC has been extensively investigated. *EPHA3* overexpression has been correlated with a poor prognosis (14, 98), which appears to occur through *EPHA3* regulation of oncogenic pathways (MAPK and VEGF) that induce cell proliferation, invasion, and angiogenesis (78, 98) (**Figure 1**).

### 3.4 EphA4

EphA4 activation contributes to the aggressive phenotype and therapeutic resistance in CRC. Although mutations in *EPHA4* have not caused inhibition of its kinase activity (99), previous studies have suggested a role for this receptor in disease pathogenesis. Overexpression of *EPHA4* is associated with promotion of liver metastasis (42) (**Table 1**). In support of these findings, a recent study showed that although *EPHA4* was negatively regulated in CRC samples compared to colorectal adenomas, its activation was associated with an aggressive epithelial-mesenchymal transition (EMT) phenotype (43). Indeed, activation of EphA4 reduced cadherin-E expression and controlled cell migration and invasion through PI3K/AKT, Wnt/β-catenin, and ERK1/2 signaling. This could contribute to the development of the EMT and lead to the well-reported therapeutic failure in rectal cancer after radiotherapy (100, 101).

### 3.5 EphA5

Recent evidence suggests that decreased EphA5 expression is associated with nodal metastasis, advanced TNM stages and unfavorable prognosis in CRC, implicating EphA5 as a potential tumor suppressor (44). In addition, *EFNA5* expression has been associated with inhibition of CRC cell migration and invasion, further supporting its potential role (45, 46) (**Table 1**). Given its potential role as a tumor suppressor, further studies are warranted to fully elucidate the significance of EphA5 in CRC progression and its implications for clinical management.

### 3.6 EphA6

Few studies have showed that the expression of the *EPHA6* gene expression was decreased in CRC (47, 48). Hafner et al. showed that the expression of the *EPHA6* gene expression was decreased in CRC (47, 48) (**Table 1**). In addition, *EPHA6* was found to have a significant prognostic value, allowing discrimination between high and low risk patients (102). Although mutations in EphA have been reported in various cohorts in the literature (103), occasional differences in the mutational profile of these molecules can be found between individuals of different ethnicities. Compared to Caucasian patients, *EPHA6* mutations were found only in African-American CRC (104, 105). This highlights the importance of analyzing genomic data through an ethnic lens, as different populations appear to have different expression patterns, including EphA6.

### 3.7 EphA7

Loss of *EPHA7* (49) is more frequent in advanced CRC, has been attributed to aberrant methylation of the 5’CpG island (50, 74). In this context, SNHG14, a newly discovered tumorigenic lncRNA, may inhibit *EPHA7* and contribute to disease progression (106) (**Table 2**). Regarding EphA7 protein levels, CRC patients had significantly higher protein levels than the control group (51). These differences could be explained by post-transcriptional regulation of this gene. In any case, these findings highlight a possible suppressor role of the *EPHA7* gene in CRC carcinogenesis (**Table 1**). It is worth noting that patients homozygous for the *EPHA7* rs2278107 (TT) reference allele showed lower disease control rates in response to irinotecan and oxaliplatin regimens (19). No mutations in *EPHA7* were found in 46 samples from Japanese patients with CRC (107). The expression of miR-944 and its target gene *EPHA7* was evaluated in Egyptian patients with CRC. The overexpression of *EPHA7* in serum levels was observed and justified by its association with the negative regulation of miR-944, which controls EphA7/PTEN/AKT signaling (108). The primary findings of these studies suggest that EphA7 may serve as an important tumor suppressor in CRC development, with genetic and epigenetic alterations regulating the tumor suppression.

### 3.8 EphA8

As with *EPHA7*, no mutations were found in *EPHA8* in 46 samples from Japanese patients with CRC (107), highlighting the heterogenous genetic changes of Eph family in different ethynes. Nevertheless, although they are the least studied ephrins in CRC, few studies have shown a possible loss of signaling of these EphAs during tumor progression, although high expression of *EPHA8* has recently been demonstrated in patients with lymph node metastasis, significantly associated with worse prognosis (52) (**Table 1**).

### 3.9 EphA10

*EPHA10* lacks kinase activity due to the presence of key amino acid changes in the kinase domain (109). This may explain why its expression and subsequent role in the transition from colorectal adenoma to CRC has been little studied. Although mRNA expression of *EPHA10* has been described in CRC (53, 54), these are preliminary investigations and further studies are needed to understand its role in tumor pathogenesis (**Table 1**).

## 4 EphB receptors

### 4.1 EphB1

Reduced expression of EphB1 appears to correlate with increased CRC progression. Low expression of *EPHB1* was correlated with poor cell differentiation (55). Furthermore, cancer cells with reduced EphB1 protein expression showed a higher capacity for tissue invasion (55) (**Table 1**). Interestingly, Mathot and colleagues demonstrated *in vitro* that mutations in the kinase domains of *EPHB1* were compared to EphB1-like mutations reduced ephrin-B1 induced compartmentalization (103). Since EphB receptors play an important role in controlling the position of different cell types in the crypt-villus axis of the intestinal epithelium (9), decreased of EphB receptor activity by somatic mutations could override the restrictive forces maintained between cells under normal conditions, leading to metastasis. Thus, some of the mutations found in *EPHB1* may contribute to the increased invasive capacity of cancers with mutations in the Eph receptor (103).

### 4.2 EphB2

*EPHB2* is located at 1p35-p36.1, a region frequently deleted in CRC and other cancers (110–112). In the normal intestinal epithelium, *EPHB2* appears to be the most expressed Eph receptor (47). Proper cell positioning and the maintenance of cellular architecture in the intestinal crypt depend on precise EphB2 signaling (5). Knockout mice for *EphB2* reduce the normal compartmentalization between stem cells and normal colonic epithelial cells (5, 113). This suggests that negative regulation of *EphB2* signaling in CRC may be a direct pathogenetic mechanism that leading to loss of tissue architecture and conferring tumor advantages in invasion and metastasis (5, 9) (**Table 1**). Indeed, *EphB2* can drive proliferation in the normal colon-adenoma sequence and function as a tumor suppressor in CRC (114, 115), and this because *EphB2* involves separate signaling pathways to regulate cell proliferation and cell migration (114, 115) (**Figure 1**).

Caudal type homeobox (Cdx) 1 and 2 are transcription factors important for homeostasis in the intestinal epithelium (116). Loss of Cdx expression has been associated with the development of intestinal polyps and with loss of the EphB1 expression. Cdx expression affects in the Notch pathway, which downregulates *EPHB1*, suggesting that the Notch-dependent signaling may be associated with the early stages of carcinogenesis in CRC (117) (**Figure 1**). Indeed, the expression of EphB2 and ephrin-B2 has been described in cell lines and CRC tissue samples (17, 118–120). In animal models, the mechanism by which EphB2 acts in CRC has become clearer. Although higher expression tended to be most evident in colon progenitor cells, it tended to decrease with disease progression (60). Loss of EphB2 has already been shown to correlate with reduced tumor growth (119, 121), CRC progression, and adverse patient outcomes (56, 122), and promotion of liver metastasis (42). Overexpression of *EPHB2* gene and EphB2 protein has been correlated with improved survival in affected patients (118, 123, 124).

Considering these findings, EphB2 may play a suppressive role in CRC development. In a study regarding the role of EphB2 in normal and neoplastic intestinal tissues, Lugli and colleagues analyzed EphB2 protein expression in more than 6,000 samples. Overall, normal colonic mucosa showed a high level of EphB2 expression, which was maintained in colonic adenomas and significantly lower in CRC, suggesting that loss of EphB2 expression accompanies the progression of colonic neoplasms. Indeed, in this significant cohort, loss of EphB2 expression correlated with an unfavorable tumor phenotype, with advanced tumor stage, high grade, presence of vascular invasion, infiltrative tumor growth, nodal metastasis, and poor survival (125).

The suppressive function of EphB2 is mainly due to its regulation of cell migration and compartmentalization (9). *EPHB2* is an important target of the Wnt/β-catenin signaling pathway, which is hyperactivated by genetic defects associated with most CRCs (5, 78). Deactivation of the adenomatous polyposis coli (APC) gene also increases of *EPHB2* expression (126). Since mutations in components of the Wnt pathway (such as in APC or β-catenin genes) contribute to aberrant activation of the β-catenin/T-cell factor-4 (TCF-4) complex that initiates most CRC (127) (**Figure 1**). *EPHB2* would be overexpressed in CRCs. In contrast, despite the maintenance of overt nuclear expression of β-catenin, *EPHB2* expression was lost in the adenoma-CRC transition. While investigating the molecular mechanisms responsible for *EPHB2* inactivation in CRC, some authors have indicated that reduced EphB2 expression in a subset of CRC cases could be attributed to DNA methylation (47, 70, 71). However, it’s worth noting that other authors do not consider this phenomenon as a common event in CRC (128, 129) (**Table 2**). Some of them believe that c-Rel, a regulator of the innate and adaptive immune response, acts as a transcriptional repressor of *EPHB2* (128).

Interestingly, Kaidi and colleagues showed that the existence of a secondary *EPHB2* silencing mechanism could be triggered by changes in the TME. Thus, hypoxia could reduce β-catenin/TCF-4 activity in animal models, resulting in decreased EphB2 expression within hypoxic regions in advanced CRC. This scenario would result from the inhibition of TCF-4 activity during hypoxia as a consequence of the direct dynamic competition between TCF-4 and HIF-1α for binding to nuclear β-catenin maintained in these tumors (130). Conversely, other authors demonstrated that the average expression of EphB2 protein in CRC was more frequently expressed in the tumor center, the most hypoxic region (131). In the context of TME, the inhibition of Eph tyrosine kinase activity or the depletion of the Eph ligand *EfnB2* has consistently led to cell death by autophagy in CRC. This cell death process can be attenuated by inhibitors of early autophagic steps, such as Spautin and 3-methyladenine, following the inhibition of phosphotyrosine-dependent Eph signaling (132).

Genetic analysis of a cohort of 50 CRC samples revealed no mutations, indicating that *EPHB2* may play a role in CRC development but is not a classical tumor suppressor gene (133). In a study of mutations in the A9 region of *EPHB2* and microsatellite instability in 481 CRC patients, Rafael and colleagues found that the risk of recurrence of tumors with high microsatellite instability carrying the *EPHB2* mutation was 3.6 times higher in carriers without a mutation (134). Decreased *EPHB2* expression in early CRC is orchestrated by several miRNAs, including miR-31-5p/miR-31-3p and miR-423-5p (38). Decreased EphB2 expression was an independent prognostic factor for recurrence and death and may have prognostic relevance in tumors with microsatellite instability (135). In a study evaluating small interfering RNA (siRNA) knockdown of ephrin-B2 in human CRC cells, some authors showed that RNA interference (RNAi) of *EFNB2* effectively silenced the *EPHB2* gene at both mRNA levels (136).

Recently, a detailed description of the EPHB2-RNF186-TAB2-TAK1 signaling cascade in CRC carcinogenesis has been described in detail (137). Upon tumor necrosis factor (TNF) stimulation, *EphB2* undergoes ubiquitination mediated by the E3 ligase RNF186, resulting in the recruitment and phosphorylation of TAB2. This interaction between TAB2 and TAK1 plays a critical role in the activation of TNF signaling. The absence of RNF186 in CRC cells significantly reduces the malignant phenotype in a murine colitis model. Furthermore, a genetic mutation in *EphB2* has been identified in a family with CRC, demonstrating a gain-of-function mutation that enhances TNF signaling activation (137).

In addition to other important contributions on the role of Eph in CRC, Batlle and his group added immensely to the scientific dialectic by describing EphB2 as an intestinal stem cell (ISC) marker. This is particularly important because EphB2 could aid in the identification of cancer stem cells and predict CRC relapse (138). In a setting where relapse and metastasis occur in a significant proportion of CRC patients, the *EphB2*-ISC relationship may be a potential therapeutic target in CRC recurrence (139). Indeed, this has motivated some studies on the effect of drugs on cancer stem cells, in which EphB2 expression correlated with drug resistance in CRC cells (140, 141).

Given that the ephrin receptor and its ligand play essential roles in intestinal mucosal renewal and that obesity-induced inflammation disrupts with ephrin signaling, Suzuki et al. (2022) investigated the role of EphB2/ephrin-B1 signaling in the development and progression of obesity-associated CRC. When obese mice were compared with control mice, ephrin-B1 expression was observed in the lower portion of the intestinal crypts in both groups, but at lower levels in the obese mice. Furthermore, a loss of EphB2 expression was observed in the carcinomas of the obese mouse model, while in control mice EphB2 was more highly expressed in the normal mucosa. Validation in human tissue samples showed similar results to the animal model. Regarding ephrin-B1, this ligand was expressed at lower levels in normal colonic mucosa of obese patients. In tumor tissue, however, higher EphB2 expression was observed in patients with BMI less than 25. These findings support the hypothesis that dysregulation of EphB2/ephrin-B1 expression promotes the development and progression of obesity-related CRC (142).

In summary, EphB2 plays a multifaceted role in CRC pathogenesis. Its decreased expression is associated with loss of tissue architecture, increased tumor invasion, and metastasis, whereas its overexpression tends to suppress tumor growth and improve patient survival. EphB2 acts as a tumor suppressor by regulating cell migration and compartmentalization, and its dysregulation is influenced by genetic mutations, epigenetic modifications, and microenvironmental factors. Targeting EphB2 signaling pathways may have therapeutic potential, particularly in the prevention of CRC recurrence and metastasis.

### 4.3 EphB3

*EPHB3* controls cell positioning and restricts cell motility (143). Indeed, the suppressor function of *EphB3* in CRC has been well documented in numerous *in vitro* and *in vivo* models (56–59). For example, in HT-29 cells, *EPHB3* overexpression increased cell-cell contact and suppressed tumor growth by reorganizing the cytoskeleton and inducing functional changes that favored mesenchymal-epithelial transition (MET)-the opposite process of EMT (57). Recently, *EPHB3* expression has been correlated with better clinical outcomes (22, 143) and longer overall survival, suggesting that EphB3 is a prognostic indicator in CRC (22) (**Table 1**).

There also seems to be a consensus that EphB3 expression is positively regulated in normal intestinal epithelial cells and colorectal adenomas but decreases in carcinomas (22, 56, 57, 72, 143). In CRC, expression decreases particularly when the tumor invades deeper tissue layers and this is even more evident in the cells that are present at the invasive front of the tumor (143). EphB3 is a direct target of Wnt/β-catenin signaling (5), which may explain the increased in the expression of this molecule in early CRC tumorigenesis.

Instead, several mechanisms have been implicated in the reduction of *EPHB3* expression in CRC. The modification of class I and III histone deacetylases and the loss of active chromatin features appear to be important in maintaining the epigenetic silencing mechanism (72) (**Table 2**). The presence of a transcriptional enhancer in the 5’ flanking region of the human *EPHB3* gene, as well as a lack of Notch activity, can also compromise the enhancer function and lead to *EPHB3* silencing (58). Induction of EMT and Snail1 represents an alternative way to deactivate the enhancer of *EPHB3* and thus lead to gene silencing (144). As recently decribed, methylation of CpG islands in the *EPHB3* promoter region in CRC cell lines appears to be critical for its epigenetic silencing (22). Furthermore, EphB receptors compartmentalize CRC cell expansion via an E-cadherin-mediated adhesion-dependent mechanism. In this sense, cells expressing EphB3 aggregate and adhere, leading to restricted dissemination of EphB-expressing tumor cells in ephrin-B1-positive territories and consequent tumor compartmentalization (9). Therefore, CRC cells could silence *EPHB3* expression to avoid repulsive interactions imposed by normal intestinal cells expressing ephrin-B1 early in tumorigenesis.

More recently, the role of *EPHB3* in the context of CRC has gained even more prominence. A major problem with targeted therapies for CRC is that in most cases, patients develop resistance to anticancer drugs (145). Increased EphB3 receptor expression leads to activation of the phosphorylation EGFR pathway and the STAT3 signaling cascade via Hedgehog (HH) signaling, which confers resistance to cetuximab in these tumors (145) (**Figure 1**). Considering this, EphB3, as well as EphA2, have become targets for cetuximab resistance studies.

In summary, epigenetic alterations can lead to changes in the expression pattern of EphB3, which correlates with tissue invasion, poorer prognosis, and drug resistance to cetuximab therapy. Therefore, targeting EphB3 signaling pathways may offer potential therapeutic strategies to overcome drug resistance in CRC treatment.

### 4.4 EphB4

Studies on the role of EphB4 in CRC have yielded conflicting results. Increased *EPHB4* gene expression suggests an intrinsic role of EphB4 in the development of a more aggressive tumor phenotype in CRC (15, 60) (**Table 1**). Indeed, EphB4 has been described as a tumor promoter associated with proliferation, invasion, and angiogenesis (146). Overexpression of *EphB4* appears to increase the migratory ability of CRC cells, leading to an increased rate of metastasis (60). For example, overexpression of EphB4 and ephrin-B2 has been reported in CRC on the luminal surface of intestinal epithelium, a cell layer known for its potential to induce metastasis (15, 17). On the other hand, contrary findings show that the expression of *EPHB4* is often reduced or lost in colorectal tumors (56, 61). Thus, *EPHB4* would have suppressive activity by limiting tumor expansion and dissemination of malignant CRC cells (56, 61). Thus, loss or decreased expression of *EphB4* in CRC would increase invasion (62), and be reflected in a worse prognosis (61) (**Table 2**). In a recent study, researchers discovered that ephrin-B2 expression was specifically increased in CRC liver metastases, while it remained unchanged in lung metastases and primary CRC tumors. Furthermore, they identified the *EFNB2/EPHB4* axis as a key player in promoting cholesterol uptake through LDLR and facilitating the colonization and growth of hepatic metastatic CRC (147). These findings provide valuable insight into the involvement of the *EFNB2/EPHB4* axis in CRC liver metastasis and highlight its potential as a promising therapeutic target.

Other conflicting data on EphB4 expression in CRC cells, particularly regarding the role of EphB4 in blood vessel formation, can also be found in the literature. Although some studies show that EphB4 does not lead to significant changes in the tumor vasculature (61, 62), EphB4 may regulate vascularization and angiogenesis (148). Indeed, inhibition of EphB4 can disrupt endothelial cell migration, vessel formation, and their branching in CRC (60, 148). Although EphB4 overexpression can enhance tumor angiogenesis, the newly formed vascular network is usually dysfunctional and inefficient, unable to meet the demands of cell proliferation. Therefore, there is a marked reduction in tumor growth (17, 119). Importantly, although EphB4-induced neoangiogenesis does not appear to be important for tumor progression, its expression may confer important migratory capabilities to CRC cells (149). In subcutaneous models, tumors induced by cells with high expression of EphB4, which had increased and enhanced vascularity, had the advantage to metastasize more easily (149).

Regarding treatment, the anti-VEGF monoclonal antibody bevacizumab was approved in 2004 as a first-line treatment for metastatic CRC in combination with chemotherapy (150). In this context, EphB4 was proposed in 2014 as a potential predictive biomarker of response in CRC patients treated with bevacizumab. Patients with high levels of *EPHB4* would have a lower response to bevacizumab as well as worse survival and could therefore benefit from more aggressive treatment (151).

### 4.5 EphB6

The role of EphB6 in CRC remains unclear and controversial. *EphB6* lacks kinase activity due to the presence of key amino acid changes in the kinase domain (152), and perhaps this may explain why its role in CRC has only been briefly investigated. The available studies on its role and the mechanisms underlying its action in CRC remain unclear and often controversial. The overexpression of EphB6 in colon tumor tissues (benign and malignant) has been demonstrated previously (63). Its expression *in vitro* promoted the proliferation of colorectal epithelial cells. Interestingly, genetic analyses showed that when EphB6 overexpression is accompanied by mutations in the APC gene, the tumor gains significant advantages in cell proliferation, invasion, and metastasis. In another *in vitro* study, EphB6 promoted the proliferation and invasion of immortalized epithelial cell lines. EphB6 showed a stronger oncogenic activity compared to EphA3, which may be related to the malignant transformation of colorectal epithelial cells (40) (**Table 1**). The authors showed that *PI3K-AKT* and *MAPK* signaling were the main pathways associated with the oncogenic activity of EphA3 and EphB6 (40) (**Figure 1**). On the other hand, using an EphB6 knockout mouse model, Mateo-Lozano and colleagues found that although loss of EphB6 correlated with lack of intestinal tumorigenesis, adenoma-carcinoma transition, and regulation of cell growth, knockout EphB6 mice exhibited an increased number of metastatic lesions (64). Consistent with these findings, Peng and colleagues showed that reduced levels of EphB6 were associated with advanced stages of disease and therefore correlated with poor prognosis (65).

## 5 Clinical trials

Despite decades of investigation into Eph receptors, there remains a paucity of clinical trials using Eph receptors to treat CRC patients. Only nine trials have evaluated drugs targeting Eph receptors in various solid malignancies, cardiac diseases, and related syndromes. These studies have used a variety of therapies, including dasatinib, MEDI-547, DS-8895a, BT5528, MM-310, EphA2-targeting DOPC-encapsulated siRNA, vaccines, and CAR-T cell immunotherapy. However, only a small number of EphA2-targeted therapies have shown promising clinical results (153). Only two studies have evaluated Eph in CRC. A phase I study in nine advanced EphA2-positive cancers, including two CRCs, evaluated the dose escalation and biodistribution of DS-8895a, an anti-EphA2 antibody (154). The results indicated that DS-8895a was well tolerated at the doses evaluated but had limited therapeutic efficacy. As a result, clinical development of DS-8895a was discontinued due to the biodistribution results and lack of response (154). The second study evaluated JI-101, a multi-kinase inhibitor including EphB4, as a potential anticancer agent. In this Phase I and II study, JI-101 was combined with everolimus, a signal transduction inhibitor that selectively inhibits mTOR, in patients with CRC, endocrine tumors and ovarian cancer. Unfortunately, the results were not published, and the trial was terminated due to lack of efficacy (NCT01149434).

## 6 Conclusions

Given the diversity of Eph expression in normal intestinal epithelial cells and the controversial role of Eph in CRC, further studies are needed to define the actual role of Eph-ephrin interaction in CRC development. Some receptors, such as the EphA3-10 and EphB5, have been little studied or remain largely unexplored in these tumors. According to the literature, it seems correct to state that Eph contributes pleiotropically to the pathogenesis of CRC. Thus, although they may have functions compatible with tumor suppression in these tumors, this does not prevent these factors from simultaneously promoting other features that may contribute to tumor promotion. However, the functional causes for EphA and EphB to alternate between tumor suppressor and tumor promoter functions are still a mystery. Clinical trials of these molecules in CRC are still in their infancy, due to ambiguous their role in this tumor. Finally, with advances in precision oncology, there is a need to identify additional targetable susceptibilities, and the Eph-ephrin system may be considered a promising therapeutic strategy when tailored to patient-specific characteristics.

## Author contributions

JFS: Conceptualization, Supervision, Writing – original draft, Writing – review & editing, Data curation. MWAG: Writing – original draft, Writing – review & editing. RdLS: Writing – original draft, Writing – review & editing. LL: Writing – original draft, Writing – review & editing. TdCK: Writing – original draft, Writing – review & editing. CCY: Writing – original draft, Writing – review & editing. AA: Conceptualization, Data curation, Supervision, Writing – review & editing. FVM: Conceptualization, Data curation, Supervision, Writing – review & editing. HPS: Conceptualization, Data curation, Supervision, Writing – review & editing. GCF: Conceptualization, Data curation, Supervision, Writing – review & editing. ESAE: Conceptualization, Data curation, Supervision, Writing – original draft, Writing – review & editing.

## References

[1] Sung H Ferlay J Siegel RL Laversanne M Soerjomataram I Jemal A. Global cancer statistics 2020: GLOBOCAN estimates of incidence and mortality worldwide for 36 cancers in 185 countries. CA Cancer J Clin. (2021) 71(3):209–49. doi: 10.3322/caac.21660 33538338

[2] Siegel RL Miller KD Wagle NS Jemal A. Cancer statistics, 2023. CA Cancer J Clin. (2023) 73:17–48. doi: 10.3322/caac.21763 36633525

[3] Pasquale EB. Eph-ephrin bidirectional signaling in physiology and disease. Cell. (2008) 133:38–52. doi: 10.1016/j.cell.2008.03.011 18394988

[4] Kosinski C Li VSW Chan ASY Zhang J Ho C Tsui WY. Gene expression patterns of human colon tops and basal crypts and BMP antagonists as intestinal stem cell niche factors. Proc Natl Acad Sci. (2007) 104:15418–23. doi: 10.1073/pnas.0707210104 PMC2000506 17881565

[5] Batlle E Henderson JT Beghtel H van den Born MMW Sancho E Huls G. β-catenin and TCF mediate cell positioning in the intestinal epithelium by controlling the expression of ephB/ephrinB. Cell. (2002) 111:251–63. doi: 10.1016/S0092-8674(02)01015-2 12408869

[6] Holmberg J Genander M Halford MM Annerén C Sondell M Chumley MJ. EphB receptors coordinate migration and proliferation in the intestinal stem cell niche. Cell. (2006) 125:1151–63. doi: 10.1016/j.cell.2006.04.030 16777604

[7] Sancho E Batlle E Clevers H. Live and let die in the intestinal epithelium. Curr Opin Cell Biol. (2003) 15:763–70. doi: 10.1016/j.ceb.2003.10.012 14644203

[8] Pasquale EB. Eph receptors and ephrins in cancer progression. Nat Rev Cancer. (2024) 24:5–27. doi: 10.1038/s41568-023-00634-x 37996538 PMC11015936

[9] Cortina C Palomo-Ponce S Iglesias M Fernández-Masip JL Vivancos A Whissell G. EphB–ephrin-B interactions suppress colorectal cancer progression by compartmentalizing tumor cells. Nat Genet. (2007) 39:1376–83. doi: 10.1038/ng.2007.11 17906625

[10] Herath NI Boyd AW. The role of Eph receptors and ephrin ligands in colorectal cancer. Int J Cancer. (2010) 126(9):2003–11. doi: 10.1002/ijc.25147 20039322

[11] Clevers H Batlle E. EphB/ephrinB receptors and wnt signaling in colorectal cancer. Cancer Res. (2006) 66:2–5. doi: 10.1158/0008-5472.CAN-05-3849 16397205

[12] Anderton M van der Meulen E Blumenthal MJ Schäfer G. The role of the eph receptor family in tumorigenesis. Cancers (Basel). (2021) 13:206. doi: 10.3390/cancers13020206 33430066 PMC7826860

[13] Brantley DM Cheng N Thompson EJ Lin Q Brekken RA Thorpe PE. Soluble Eph A receptors inhibit tumor angiogenesis and progression. vivo Oncogene. (2002) 21:7011–26. doi: 10.1038/sj.onc.1205679 12370823

[14] Xi H-Q Zhao P. Clinicopathological significance and prognostic value of EphA3 and CD133 expression in colorectal carcinoma. J Clin Pathol. (2011) 64:498–503. doi: 10.1136/jcp.2010.087213 21415057

[15] Stephenson SA Slomka S Douglas EL Hewett PJ Hardingham JE. Receptor protein tyrosine kinase EphB4 is up-regulated in colon cancer. BMC Mol Biol. (2001) 2:15. doi: 10.1186/1471-2199-2-15 11801186 PMC64642

[16] Kataoka H Igarashi H Kanamori M Ihara M Wang J-D Wang Y-J. Correlation of EPHA2 overexpression with high microvessel count in human primary colorectal cancer. Cancer Sci. (2004) 95:136–41. doi: 10.1111/j.1349-7006.2004.tb03194.x PMC11159731 14965363

[17] Liu W Ahmad SA Jung YD Reinmuth N Fan BSF Bucana CD. Coexpression of ephrin-Bs and their receptors in colon carcinoma. Cancer. (2002) 94:934–9. doi: 10.1002/cncr.10122 11920461

[18] Phan NN Liu S Wang C-Y Hsu H-P Lai M-D Li C-Y. Overexpressed gene signature of EPH receptor A/B family in cancer patients-comprehensive analyses from the public high-throughput database. Int J Clin Exp Pathol. (2020) 13(5):1220–42. PMC7270671 32509099

[19] Kim JC Kim SY Cho DH Roh SA Choi EY Jo YK. Genome-wide identification of chemosensitive single nucleotide polymorphism markers in colorectal cancers. Cancer Sci. (2010) 101:1007–13. doi: 10.1111/j.1349-7006.2009.01461.x PMC11159617 20085586

[20] Dong Y Wang J Sheng Z Li G Ma H Wang X. Downregulation of EphA1 in colorectal carcinomas correlates with invasion and metastasis. Modern Pathol. (2009) 22:151–60. doi: 10.1038/modpathol.2008.188 19011600

[21] Xi H-Q Wu X-S Wei B Chen L. Eph receptors and ephrins as targets for cancer therapy. J Cell Mol Med. (2012) 16:2894–909. doi: 10.1111/j.1582-4934.2012.01612.x PMC4393718 22862837

[22] Xuan Z Huang J Gao L Wang Y Wang J Sun Y. Receptor tyrosine kinase ephB3: a prognostic indicator in colorectal carcinoma. Pathol Oncol Res. (2020) 26:541–9. doi: 10.1007/s12253-018-0562-x 30535864

[23] Eph Nomenclature Committee. Unified nomenclature for eph family receptors and their ligands, the ephrins. Cell. (1997) 90:403–4. doi: 10.1016/S0092-8674(00)80500-0 9267020

[24] Gale NW Holland SJ Valenzuela DM Flenniken A Pan L Ryan TE. Eph receptors and ligands comprise two major specificity subclasses and are reciprocally compartmentalized during embryogenesis. Neuron. (1996) 17:9–19. doi: 10.1016/S0896-6273(00)80276-7 8755474

[25] Himanen J-P Rajashankar KR Lackmann M Cowan CA Henkemeyer M Nikolov DB. Crystal structure of an Eph receptor–ephrin complex. Nature. (2001) 414:933–8. doi: 10.1038/414933a 11780069

[26] Labrador JP Brambilla R Klein R. The N-terminal globular domain of Eph receptors is sufficient for ligand binding and receptor signaling. EMBO J. (1997) 16:3889–97. doi: 10.1093/emboj/16.13.3889 PMC1170013 9233799

[27] Arvanitis D Davy A. Eph/ephrin signaling: networks. Genes Dev. (2008) 22:416–29. doi: 10.1101/gad.1630408 PMC2731651 18281458

[28] Brass LF Newman DK Wannermacher KM Zhu L Stalker TJ. “Signal Transduction During Platelet Plug Formation.,”. In: Platelets. Elsevier (2013). p. 367–98. doi: 10.1016/B978-0-12-387837-3.00019-5

[29] Dai D Huang Q Nussinov R Ma B. Promiscuous and specific recognition among ephrins and Eph receptors. Biochim Biophys Acta (BBA) - Proteins Proteomics. (2014) 1844:1729–40. doi: 10.1016/j.bbapap.2014.07.002 PMC4157952 25017878

[30] Darling TK Lamb TJ. Emerging roles for eph receptors and ephrin ligands in immunity. Front Immunol. (2019) 10:1473. doi: 10.3389/fimmu.2019.01473 31333644 PMC6620610

[31] Murai KK Pasquale EB. `Eph’ective signaling: forward, reverse and crosstalk. J Cell Sci. (2003) 116:2823–32. doi: 10.1242/jcs.00625 12808016

[32] Wu Y Du Z Mou J Qiu X Chen J Cai S. The functions of ephA1 receptor tyrosine kinase in several tumors. Curr Med Chem. (2023) 30:2340–53. doi: 10.2174/0929867329666220820125638 35996244

[33] Asadian F Ghadyani M Antikchi MH Dastgheib SA Neamatzadeh H Sheikhpour E. Association of epidermal growth factor 61A>G, survivin -31G>C, and EFNA1 -1732G>A polymorphisms with susceptibility to colorectal cancer. J Gastrointest Cancer. (2022) 53:78–83. doi: 10.1007/s12029-020-00551-4 33180239

[34] Liu Z Weng S Dang Q Xu H Ren Y Guo C. Gene interaction perturbation network deciphers a high-resolution taxonomy in colorectal cancer. Elife. (2022) 11:e81114. doi: 10.7554/eLife.81114 36345721 PMC9643007

[35] Salem E Keshvari A Mahdavinezhad A Soltanian AR Saidijam M Afshar S. Role of EFNA1 SNP (rs12904) in tumorigenesis and metastasis of colorectal cancer: A bioinformatic analysis and HRM SNP genotyping verification. Asian Pacific J Cancer Prev. (2022) 23:3523–31. doi: 10.31557/APJCP.2022.23.10.3523 PMC9924350 36308379

[36] Herath NI Doecke J Spanevello MD Leggett BA Boyd AW. Epigenetic silencing of EphA1 expression in colorectal cancer is correlated with poor survival. Br J Cancer. (2009) 100:1095–102. doi: 10.1038/sj.bjc.6604970 PMC2670002 19277044

[37] Shi L Itoh F Itoh S Takahashi S Yamamoto M Kato M. Ephrin-A1 promotes the Malignant progression of intestinal tumors in Apcmin/+ mice. Oncogene. (2008) 27:3265–73. doi: 10.1038/sj.onc.1210992 18246128

[38] De Robertis M Mazza T Fusilli C Loiacono L Poeta ML Sanchez M. EphB2 stem-related and EphA2 progression-related miRNA-based networks in progressive stages of CRC evolution: clinical significance and potential miRNA drivers. Mol Cancer. (2018) 17:169. doi: 10.1186/s12943-018-0912-z 30501625 PMC6271583

[39] Saito T Masuda N Miyazaki T Kanoh K Suzuki H Shimura T. Expression of EphA2 and E-cadherin in colorectal cancer: Correlation with cancer metastasis. Oncol Rep. (2004) 11(3):605–11. doi: 10.3892/or.11.3.605 14767510

[40] Wang J Zhang Y Ma J Yang C Wang M Lv J. Determining the effects of Ephrin Type B Receptor 6 and Type A Receptor 3 on facilitating colorectal epithelial cell Malignant transformation. Neoplasma. (2021) 68:955–64. doi: 10.4149/neo_2021_210309N304 34196214

[41] Wang Y Xuan Z Wang B Zhang D Zhang C Wang J. EphA3 downregulation by hypermethylation associated with lymph node metastasis and TNM stage in colorectal cancer. Dig Dis Sci. (2019) 64:1514–22. doi: 10.1007/s10620-018-5421-9 30560328

[42] Oshima T Akaike M Yoshihara K Shiozawa M Yamamoto N Sato T. Overexpression of EphA4 gene and reduced expression of EphB2 gene correlates with liver metastasis in colorectal cancer. Int J Oncol. (2008) 33(3):573–7. 18695888

[43] Hauptman N Boštjančič E Žlajpah M Ranković B Zidar N. Bioinformatics analysis reveals most prominent gene candidates to distinguish colorectal adenoma from adenocarcinoma. BioMed Res Int. (2018) 2018:1–10. doi: 10.1155/2018/9416515 PMC6106857 30175151

[44] Gu S Feng J Jin Q Wang W Zhang S. Reduced expression of EphA5 is associated with lymph node metastasis, advanced TNM stage, and poor prognosis in colorectal carcinoma. Histol Histopathol. (2017) 32:491–7. doi: 10.14670/HH-11-815 27651378

[45] Li S Hou X Wu C Han L Li Q Wang J. MiR-645 promotes invasiveness, metastasis and tumor growth in colorectal cancer by targeting EFNA5. Biomed Pharmacother. (2020) 125:109889. doi: 10.1016/j.biopha.2020.109889 32036212

[46] Wang T-H Chang J-L Ho J-Y Wu H-C Chen T-C. EphrinA5 suppresses colon cancer development by negatively regulating epidermal growth factor receptor stability. FEBS J. (2012) 279:251–63. doi: 10.1111/j.1742-4658.2011.08419.x 22074469

[47] Hafner C Schmitz G Meyer S Bataille F Hau P Langmann T. Differential gene expression of eph receptors and ephrins in benign human tissues and cancers. Clin Chem. (2004) 50:490–9. doi: 10.1373/clinchem.2003.026849 14726470

[48] Huang H Xu S Chen A Li F Wu J Tu X. Identification of a 5-gene-based scoring system by WGCNA and LASSO to predict prognosis for rectal cancer patients. Analytical Cell Pathol. (2021) 2021:1–17. doi: 10.1155/2021/6697407 PMC8012151 33833937

[49] Kober P Bujko M Olędzki J Tysarowski A Siedlecki JA. Methyl-CpG binding column-based identification of nine genes hypermethylated in colorectal cancer. Mol Carcinog. (2011) 50:846–56. doi: 10.1002/mc.20763 21438024

[50] Wang J Kataoka H Suzuki M Sato N Nakamura R Tao H. Downregulation of EphA7 by hypermethylation in colorectal cancer. Oncogene. (2005) 24:5637–47. doi: 10.1038/sj.onc.1208720 16007213

[51] Üçüncü M Serilmez M Sarı M Bademler S Karabulut S. The diagnostic significance of PDGF, ephA7, CCR5, and CCL5 levels in colorectal cancer. Biomolecules. (2019) 9:464. doi: 10.3390/biom9090464 31505877 PMC6770732

[52] Li X Zhang Q Zhao L Jiang L Qi A Wei Q. A combined four-mRNA signature associated with lymphatic metastasis for prognosis of colorectal cancer. J Cancer. (2020) 11:2139–49. doi: 10.7150/jca.38796 PMC7052913 32127941

[53] Nagano K Yamashita T Inoue M Higashisaka K Yoshioka Y Abe Y. Eph receptor A10 has a potential as a target for a prostate cancer therapy. Biochem Biophys Res Commun. (2014) 450:545–9. doi: 10.1016/j.bbrc.2014.06.007 24924629

[54] Shin W Park MK Lee YH Kim KW Lee H Lee S. The catalytically defective receptor protein tyrosine kinase EphA10 promotes tumorigenesis in pancreatic cancer cells. Cancer Sci. (2020) 111:3292–302. doi: 10.1111/cas.14568 PMC7469775 32644283

[55] Sheng Z Wang J Dong Y Ma H Zhou H Sugimura H. EphB1 is underexpressed in poorly differentiated colorectal cancers. Pathobiology. (2008) 75:274–80. doi: 10.1159/000151707 18931529

[56] Batlle E Bacani J Begthel H Jonkeer S Gregorieff A van de Born M. EphB receptor activity suppresses colorectal cancer progression. Nature. (2005) 435:1126–30. doi: 10.1038/nature03626 15973414

[57] Chiu S-T Chang K-J Ting C-H Shen H-C Li H Hsieh F-J. Over-expression of EphB3 enhances cell–cell contacts and suppresses tumor growth in HT-29 human colon cancer cells. Carcinogenesis. (2009) 30:1475–86. doi: 10.1093/carcin/bgp133 19483190

[58] Jägle S Rönsch K Timme S Andrlová H Bertrand M Jäger M. Silencing of the EPHB3 tumor-suppressor gene in human colorectal cancer through decommissioning of a transcriptional enhancer. Proc Natl Acad Sci. (2014) 111:4886–91. doi: 10.1073/pnas.1314523111 PMC3977263 24707046

[59] Murphy M Chatterjee SS Jain S Katari M DasGupta R. TCF7L1 modulates colorectal cancer growth by inhibiting expression of the tumor-suppressor gene EPHB3. Sci Rep. (2016) 6:28299. doi: 10.1038/srep28299 27333864 PMC4917863

[60] Kumar SR Scehnet JS Ley EJ Singh J Krasnoperov V Liu R. Preferential induction of ephB4 over ephB2 and its implication in colorectal cancer progression. Cancer Res. (2009) 69:3736–45. doi: 10.1158/0008-5472.CAN-08-3232 19366806

[61] Davalos V Dopeso H Castaño J Wilson AJ Vilardell F Romero-Gimenez J. EPHB4 and survival of colorectal cancer patients. Cancer Res. (2006) 66:8943–8. doi: 10.1158/0008-5472.CAN-05-4640 16982731

[62] Dopeso H Mateo-Lozano S Mazzolini R Rodrigues P Lagares-Tena L Ceron J. The receptor tyrosine kinase EPHB4 has tumor suppressor activities in intestinal tumorigenesis. Cancer Res. (2009) 69:7430–8. doi: 10.1158/0008-5472.CAN-09-0706 19738063

[63] Xu D Yuan L Liu X Li M Zhang F Gu X. EphB6 overexpression and Apc mutation together promote colorectal cancer. Oncotarget. (2016) 7:31111–21. doi: 10.18632/oncotarget.9080 PMC5058743 27145271

[64] Mateo-Lozano S Bazzocco S Rodrigues P Mazzolini R Andretta E Dopeso H. Loss of the EPH receptor B6 contributes to colorectal cancer metastasis. Sci Rep. (2017) 7:43702. doi: 10.1038/srep43702 28262839 PMC5337985

[65] Peng L Tu P Wang X Shi S Zhou X Wang J. Loss of EphB6 protein expression in human colorectal cancer correlates with poor prognosis. J Mol Histol. (2014) 45:555–63. doi: 10.1007/s10735-014-9577-0 24912672

[66] Wu BO Jiang WG Zhou D Cui Y-X. Knockdown of EPHA1 by CRISPR/CAS9 promotes adhesion and motility of HRT18 colorectal carcinoma cells. Anticancer Res. (2016) 36(3):1211–9. 26977017

[67] Potla L Boghaert ER Armellino D Frost P Damle NK. Reduced expression of EphrinA1 (EFNA1) inhibits three-dimensional growth of HT29 colon carcinoma cells. Cancer Lett. (2002) 175:187–95. doi: 10.1016/S0304-3835(01)00613-9 11741747

[68] Yamamoto H Tei M Uemura M Takemasa I Uemura Y Murata K. Ephrin-A1 mRNA is associated with poor prognosis of colorectal cancer. Int J Oncol. (2013) 42:549–55. doi: 10.3892/ijo.2012.1750 23258614

[69] Ieguchi K Tomita T Takao T Omori T Mishima T Shimizu I. Analysis of ADAM12-mediated ephrin-A1 cleavage and its biological functions. Int J Mol Sci. (2021) 22:2480. doi: 10.3390/ijms22052480 33804570 PMC7957476

[70] Alazzouzi H Davalos V Kokko A Domingo E Woerner SM Wilson AJ. Mechanisms of inactivation of the receptor tyrosine kinase EPHB2 in colorectal tumors. Cancer Res. (2005) 65:10170–3. doi: 10.1158/0008-5472.CAN-05-2580 16288001

[71] Dong L Ma L Ma GH Ren H. Genome-wide analysis reveals DNA methylation alterations in obesity associated with high risk of colorectal cancer. Sci Rep. (2019) 9:5100. doi: 10.1038/s41598-019-41616-0 30911103 PMC6433909

[72] Rönsch K Jäger M Schöpflin A Danciu M Laßmann S Hecht A. Class I and III HDACs and loss of active chromatin features contribute to epigenetic silencing of CDX1 and EPHB tumor suppressor genes in colorectal cancer. Epigenetics. (2011) 6:610–22. doi: 10.4161/epi.6.5.15300 21393996

[73] Bogan C Chen J O’Sullivan MG Cormier RT. Loss of EphA2 receptor tyrosine kinase reduces Apc min/+ tumorigenesis. Int J Cancer. (2009) 124:1366–71. doi: 10.1002/ijc.24083 19089910

[74] Herath NI Spanevello MD Doecke JD Smith FM Pouponnot C Boyd AW. Complex expression patterns of Eph receptor tyrosine kinases and their ephrin ligands in colorectal carcinogenesis. Eur J Cancer. (2012) 48:753–62. doi: 10.1016/j.ejca.2011.07.003 21852108

[75] Walkiewicz K Kozieł P Bednarczyk M Błażelonis A Mazurek U Muc-Wierzgoń M. Expression of migration-related genes in human colorectal cancer and activity of a disintegrin and metalloproteinase 17. BioMed Res Int. (2016) 2016:1–5. doi: 10.1155/2016/8208904 PMC4826671 27110571

[76] Baeten CIM Hillen F Pauwels P de Bruine AP Baeten CGMI. Prognostic role of vasculogenic mimicry in colorectal cancer. Dis Colon Rectum. (2009) 52:2028–35. doi: 10.1007/DCR.0b013e3181beb4ff 19934926

[77] Dunne PD Dasgupta S Blayney JK McArt DG Redmond KL Weir J-A. EphA2 expression is a key driver of migration and invasion and a poor prognostic marker in colorectal cancer. Clin Cancer Res. (2016) 22:230–42. doi: 10.1158/1078-0432.CCR-15-0603 PMC4694030 26283684

[78] Lau A Le N Nguyen C Kandpal RP. Signals transduced by Eph receptors and ephrin ligands converge on MAP kinase and AKT pathways in human cancers. Cell Signal. (2023) 104:110579. doi: 10.1016/j.cellsig.2022.110579 36572189

[79] Arabzadeh A McGregor K Breton V van der Kraak L Akavia UD Greenwood CMT. EphA2 signaling is impacted by carcinoembryonic antigen cell adhesion molecule 1-L expression in colorectal cancer liver metastasis in a cell context-dependent manner. Oncotarget. (2017) 8:104330–46. doi: 10.18632/oncotarget.22236 PMC5732810 29262644

[80] Li W Wu C-C Wang S Zhou L Qiao L Ba W. Identification of the target protein of the metastatic colorectal cancer-specific aptamer W3 as a biomarker by aptamer-based target cells sorting and functional characterization. Biosens Bioelectron. (2022) 213:114451. doi: 10.1016/j.bios.2022.114451 35700603

[81] Grassilli E Cerrito MG. Emerging actionable targets to treat therapy-resistant colorectal cancers. Cancer Drug Resist. (2022) 5(1):36–63. doi: 10.20517/cdr.2021.96 PMC8992594 35582524

[82] Martini G Cardone C Vitiello PP Belli V Napolitano S Troiani T. EPHA2 is a predictive biomarker of resistance and a potential therapeutic target for improving antiepidermal growth factor receptor therapy in colorectal cancer. Mol Cancer Ther. (2019) 18:845–55. doi: 10.1158/1535-7163.MCT-18-0539 30824612

[83] Strimpakos A Pentheroudakis G Kotoula V De Roock W Kouvatseas G Papakostas P. The prognostic role of ephrin A2 and endothelial growth factor receptor pathway mediators in patients with advanced colorectal cancer treated with cetuximab. Clin Colorectal Cancer. (2013) 12:267–274.e2. doi: 10.1016/j.clcc.2013.07.001 24050852

[84] Fornasier G Francescon S Baldo P. An update of efficacy and safety of cetuximab in metastatic colorectal cancer: A narrative review. Adv Ther. (2018) 35:1497–509. doi: 10.1007/s12325-018-0791-0 30218345

[85] De Robertis M Loiacono L Fusilli C Poeta ML Mazza T Sanchez M. Dysregulation of EGFR pathway in ephA2 cell subpopulation significantly associates with poor prognosis in colorectal cancer. Clin Cancer Res. (2017) 23:159–70. doi: 10.1158/1078-0432.CCR-16-0709 PMC5822042 27401248

[86] Cioce M Fazio VM. EphA2 and EGFR: friends in life, partners in crime. Can ephA2 be a predictive biomarker of response to anti-EGFR agents? Cancers (Basel). (2021) 13:700. doi: 10.3390/cancers13040700 33572284 PMC7915460

[87] Cuyàs E Queralt B Martin-Castillo B Bosch-Barrera J Menendez JA. EphA2 receptor activation with ephrin-A1 ligand restores cetuximab efficacy in NRAS-mutant colorectal cancer cells. Oncol Rep. (2017) 38:263–70. doi: 10.3892/or.2017.5682 28560458

[88] Yamaguchi S Tatsumi T Takehara T Sasakawa A Yamamoto M Kohga K. EphA2-derived peptide vaccine with amphiphilic poly(γ-glutamic acid) nanoparticles elicits an anti-tumor effect against mouse liver tumor. Cancer Immunol Immunother. (2010) 59:759–67. doi: 10.1007/s00262-009-0796-2 PMC11030875 19943047

[89] Yamaguchi S Tatsumi T Takehara T Sakamori R Uemura A Mizushima T. Immunotherapy of murine colon cancer using receptor tyrosine kinase EphA2-derived peptide-pulsed dendritic cell vaccines. Cancer. (2007) 110:1469–77. doi: 10.1002/cncr.22958 17685394

[90] Harly C Joyce SP Domblides C Bachelet T Pitard V Mannat C. Human γδ T cell sensing of AMPK-dependent metabolic tumor reprogramming through TCR recognition of EphA2. Sci Immunol. (2021) 6(61):eaba9010. doi: 10.1126/sciimmunol.aba9010 34330813

[91] Tröster A DiPrima M Jores N Kudlinzki D Sreeramulu S Gande SL. Optimization of the lead compound NVP-BHG712 as a colorectal cancer inhibitor. Chem – A Eur J. (2023) 29(23):e202203967. doi: 10.1002/chem.202203967 PMC10133194 36799129

[92] Torlot L Jarzab A Albert J Pók-Udvari Á Stahler A Holch JW. Proteomics uncover EPHA2 as a potential novel therapeutic target in colorectal cancer cell lines with acquired cetuximab resistance. J Cancer Res Clin Oncol. (2023) 149:669–82. doi: 10.1007/s00432-022-04416-0 PMC9931833 36401637

[93] Andretta E Cartón-García F Martínez-Barriocanal Á de Marcondes PG Jimenez-Flores LM Macaya I. Investigation of the role of tyrosine kinase receptor EPHA3 in colorectal cancer. Sci Rep. (2017) 7:41576. doi: 10.1038/srep41576 28169277 PMC5294649

[94] Hinoue T Weisenberger DJ Pan F Campan M Kim M Young J. Analysis of the association between CIMP and BRAFV600E in colorectal cancer by DNA methylation profiling. PloS One. (2009) 4:e8357. doi: 10.1371/journal.pone.0008357 20027224 PMC2791229

[95] Bardelli A Parsons DW Silliman N Ptak J Szabo S Saha S. Mutational analysis of the tyrosine kinome in colorectal cancers. Science. (2003) 300:949. doi: 10.1126/science.1082596 12738854

[96] Lisabeth EM Fernandez C Pasquale EB. Cancer somatic mutations disrupt functions of the ephA3 receptor tyrosine kinase through multiple mechanisms. Biochemistry. (2012) 51:1464–75. doi: 10.1021/bi2014079 PMC3471792 22242939

[97] Sjoblom T Jones S Wood LD Parsons DW Lin J Barber TD. The consensus coding sequences of human breast and colorectal cancers. Sci (1979). (2006) 314:268–74. doi: 10.1126/science.1133427 16959974

[98] Li M Yang C Liu X Yuan L Zhang F Wang M. EphA3 promotes Malignant transformation of colorectal epithelial cells by upregulating oncogenic pathways. Cancer Lett. (2016) 383:195–203. doi: 10.1016/j.canlet.2016.10.004 27721017

[99] Duplaquet L Figeac M Leprêtre F Frandemiche C Villenet C Sebda S. Functional analysis of somatic mutations affecting receptor tyrosine kinase family in metastatic colorectal cancer. Mol Cancer Ther. (2019) 18:1137–48. doi: 10.1158/1535-7163.MCT-18-0582 30926633

[100] de Marcondes PG Bastos LG De-Freitas-Junior JCM Rocha MR Morgado-Díaz JA. EphA4-mediated signaling regulates the aggressive phenotype of irradiation survivor colorectal cancer cells. Tumor Biol. (2016) 37:12411–22. doi: 10.1007/s13277-016-5120-0 27323967

[101] de Marcondes PG Morgado-Díaz JA. The role of ephA4 signaling in radiation-induced EMT-like phenotype in colorectal cancer cells. J Cell Biochem. (2017) 118:442–5. doi: 10.1002/jcb.25738 27632701

[102] Zuo S Dai G Ren X. Identification of a 6-gene signature predicting prognosis for colorectal cancer. Cancer Cell Int. (2019) 19:6. doi: 10.1186/s12935-018-0724-7 30627052 PMC6321660

[103] Mathot L Kundu S Ljungström V Svedlund J Moens L Adlerteg T. Somatic ephrin receptor mutations are associated with metastasis in primary colorectal cancer. Cancer Res. (2017) 77:1730–40. doi: 10.1158/0008-5472.CAN-16-1921 28108514

[104] Carethers JM. Clinical and genetic factors to inform reducing colorectal cancer disparitites in african Americans. Front Oncol. (2018) 8:531. doi: 10.3389/fonc.2018.00531 30524961 PMC6256119

[105] Guda K Veigl ML Varadan V Nosrati A Ravi L Lutterbaugh J. Novel recurrently mutated genes in African American colon cancers. Proc Natl Acad Sci. (2015) 112:1149–54. doi: 10.1073/pnas.1417064112 PMC4313860 25583493

[106] Di W Weinan X Xin L Zhiwei Y Xinyue G Jinxue T. Long noncoding RNA SNHG14 facilitates colorectal cancer metastasis through targeting EZH2-regulated EPHA7. Cell Death Dis. (2019) 10:514. doi: 10.1038/s41419-019-1707-x 31273190 PMC6609685

[107] Shao R-X Kato N Lin L-J Muroyama R Moriyama M Ikenoue T. Absence of tyrosine kinase mutations in Japanese colorectal cancer patients. Oncogene. (2007) 26:2133–5. doi: 10.1038/sj.onc.1210007 17016444

[108] Shaker OG Ayeldeen G Abdelhamid AM. Circulating microRNA-944 and its target gene EPHA7 as a potential biomarker for colorectal cancer. Arch Physiol Biochem. (2020) 128(5):1181–7. doi: 10.1080/13813455.2020.1762658 32421395

[109] Aasheim H-C Patzke S Hjorthaug HS Finne EF. Characterization of a novel Eph receptor tyrosine kinase, EphA10, expressed in testis. Biochim Biophys Acta (BBA) - Gen Subj. (2005) 1723:1–7. doi: 10.1016/j.bbagen.2005.01.011 15777695

[110] Praml C Finke LH Herfarth C Schlag P Schwab M Amler L. Deletion mapping defines different regions in 1p34.2-pter that may harbor genetic information related to human colorectal cancer. Oncogene. (1995) 11(7):1357–62. 7478557

[111] Ragnarsson G Eiriksdottir G Johannsdottir JT Jonasson JG Egilsson V Ingvarsson S. Loss of heterozygosity at chromosome 1p in different solid human tumours: association with survival. Br J Cancer. (1999) 79:1468–74. doi: 10.1038/sj.bjc.6690234 PMC2362732 10188892

[112] Liu W Yu C Li J Fang J. The roles of ephB2 in cancer. Front Cell Dev Biol. (2022) 10:788587. doi: 10.3389/fcell.2022.788587 35223830 PMC8866850

[113] Leung HW Leung CON Lau EY Chung KPS Mok EH Lei MML. EPHB2 activates β-catenin to enhance cancer stem cell properties and drive sorafenib resistance in hepatocellular carcinoma. Cancer Res. (2021) 81:3229–40. doi: 10.1158/0008-5472.CAN-21-0184 33903122

[114] Genander M Halford MM Xu N-J Eriksson M Yu Z Qiu Z. Dissociation of ephB2 signaling pathways mediating progenitor cell proliferation and tumor suppression. Cell. (2009) 139:679–92. doi: 10.1016/j.cell.2009.08.048 PMC2786256 19914164

[115] Noberini R Pasquale EB. Proliferation and tumor suppression: not mutually exclusive for eph receptors. Cancer Cell. (2009) 16:452–4. doi: 10.1016/j.ccr.2009.11.008 19962662

[116] Hryniuk A Grainger S Savory JGA Lohnes D. Cdx1 and cdx2 function as tumor suppressors. J Biol Chem. (2014) 289:33343–54. doi: 10.1074/jbc.M114.583823 PMC4246091 25320087

[117] Zhu Y Hryniuk A Foley T Hess B Lohnes D. Cdx2 regulates intestinal ephrinB1 through the notch pathway. Genes (Basel). (2021) 12:188. doi: 10.3390/genes12020188 33525395 PMC7911442

[118] Jang BG Kim HS Chang WY Bae JM Kang GH. Prognostic significance of EPHB2 expression in colorectal cancer progression. J Pathol Transl Med. (2018) 52:298–306. doi: 10.4132/jptm.2018.06.29 30016858 PMC6166016

[119] Liu W Jung YD Ahmad SA McCarty MF Stoeltzing O Reinmuth N. Effects of overexpression of ephrin-B2 on tumour growth in human colorectal cancer. Br J Cancer. (2004) 90:1620–6. doi: 10.1038/sj.bjc.6601723 PMC2409715 15083195

[120] Mao W Luis E Ross S Silva J Tan C Crowley C. EphB2 as a therapeutic antibody drug target for the treatment of colorectal cancer. Cancer Res. (2004) 64:781–8. doi: 10.1158/0008-5472.CAN-03-1047 14871799

[121] Senior PV Zhang BX Chan STF. Loss of cell-surface receptor EphB2 is important for the growth, migration, and invasiveness of a colon cancer cell line. Int J Colorectal Dis. (2010) 25:687–94. doi: 10.1007/s00384-010-0916-7 20339854

[122] Guo DL Zhang J Yuen ST Tsui WY Chan ASY Ho C. Reduced expression of EphB2 that parallels invasion and metastasis in colorectal tumours. Carcinogenesis. (2006) 27:454–64. doi: 10.1093/carcin/bgi259 16272170

[123] Jubb AM Zhong F Bheddah S Grabsch HI Frantz GD Mueller W. EphB2 is a prognostic factor in colorectal cancer. Clin Cancer Res. (2005) 11:5181–7. doi: 10.1158/1078-0432.CCR-05-0143 16033834

[124] Vidaurreta M Rafael S Veganzones S de la Orden V Fernández C Gómez-Casaseca R. Influence of A9 region mutation in ephB2 gene in the prognosis of patients with colorectal adenocarcinoma. Ann Surg Oncol. (2011) 18:1501–5. doi: 10.1245/s10434-010-1448-7 21161727

[125] Lugli A Spichtin H Maurer R Mirlacher M Kiefer J Huusko P. EphB2 expression across 138 human tumor types in a tissue microarray: high levels of expression in gastrointestinal cancers. Clin Cancer Res. (2005) 11:6450–8. doi: 10.1158/1078-0432.CCR-04-2458 16166419

[126] Sansom OJ. Loss of Apc *in vivo* immediately perturbs Wnt signaling, differentiation, and migration. Genes Dev. (2004) 18:1385–90. doi: 10.1101/gad.287404 PMC423189 15198980

[127] Morin PJ. Activation of beta -Catenin-Tcf Signaling in Colon Cancer by Mutations in beta -Catenin or APC. Sci (1979). (1997) 275:1787–90. doi: 10.1126/science.275.5307.1787 9065402

[128] Fu T Li P Wang H He Y Luo D Zhang A. c-Rel is a transcriptional repressor of EPHB2 in colorectal cancer. J Pathol. (2009) 219:103–13. doi: 10.1002/path.2590 19621336

[129] Wu Q Lind GE Aasheim H-C Micci F Silins I Tropé CG. The EPH receptor bs (EPHBs) promoters are unmethylated in colon and ovarian cancers. Epigenetics. (2007) 2:237–43. doi: 10.4161/epi.2.4.5406 18281782

[130] Kaidi A Moorghen M Williams AC Paraskeva C. Is the downregulation of EphB2 receptor expression during colorectal tumorigenesis due to hypoxia? Gut. (2007) 56:1637–8. doi: 10.1136/gut.2007.131540 PMC2095662 17938437

[131] Karamitopoulou E Zlobec I Panayiotides I Patsouris ES Peros G Rallis G. Systematic analysis of proteins from different signaling pathways in the tumor center and the invasive front of colorectal cancer. Hum Pathol. (2011) 42:1888–96. doi: 10.1016/j.humpath.2010.06.020 21664646

[132] DiPrima M Wang D Tröster A Maric D Terrades-Garcia N Ha T. Identification of Eph receptor signaling as a regulator of autophagy and a therapeutic target in colorectal carcinoma. Mol Oncol. (2019) 13:2441–59. doi: 10.1002/1878-0261.12576 PMC6822245 31545551

[133] Oba SM Wang Y-J Song J-P Li Z-Y Kobayashi K Tsugane S. Genomic structure and loss of heterozygosity of EPHB2 in colorectal cancer. Cancer Lett. (2001) 164:97–104. doi: 10.1016/S0304-3835(00)00716-3 11166921

[134] Rafael S Vidaurreta M Veganzones S de la Orden V Mediero B Gutierrez ML. A9 region in EPHB2 mutation is frequent in tumors with microsatellite instability. Analysis of prognosis. Anticancer Res. (2013) 33(11):5159–63. 24222164

[135] Drucker A Arnason T Yan Sen R Aljawad M Thompson K Huang W-Y. Ephrin B2 receptor and microsatellite status in lymph node-positive colon cancer survival. Transl Oncol. (2013) 6:520–7. doi: 10.1593/tlo.13385 PMC3799194 24151532

[136] Li P Chen W Wang Y Fu X Wen K Qian J. Effects of ephrinB2 gene siRNA on the biological behavior of human colorectal cancer cells. Oncol Rep. (2015) 33:758–66. doi: 10.3892/or.2014.3633 25434750

[137] Zhang H Cui Z Pan T Hu H He R Yi M. RNF186/EPHB2 axis is essential in regulating TNF signaling for colorectal tumorigenesis in colorectal epithelial cells. J Immunol. (2022) 209:1796–805. doi: 10.4049/jimmunol.2200229 PMC9553791 36130827

[138] Merlos-Suárez A Barriga FM Jung P Iglesias M Céspedes MV Rossell D. The intestinal stem cell signature identifies colorectal cancer stem cells and predicts disease relapse. Cell Stem Cell. (2011) 8:511–24. doi: 10.1016/j.stem.2011.02.020 21419747

[139] Zhang X. EphB2: a signature of colorectal cancer stem cells to predict relapse. Protein Cell. (2011) 2:347–8. doi: 10.1007/s13238-011-1058-6 PMC4875343 21667331

[140] El Khoury F Corcos L Durand S Simon B Le Jossic-Corcos C. Acquisition of anticancer drug resistance is partially associated with cancer stemness in human colon cancer cells. Int J Oncol. (2016) 49:2558–68. doi: 10.3892/ijo.2016.3725 27748801

[141] Feng Y Gao S Gao Y Wang X Chen Z. Anti-EGFR antibody sensitizes colorectal cancer stem-like cells to Fluorouracil-induced apoptosis by affecting autophagy. Oncotarget. (2016) 7:81402–9. doi: 10.18632/oncotarget.13233 PMC5348401 27833077

[142] Suzuki Y Okabayashi K Hasegawa H Tsuruta M Seishima R Tokuda T. Role of EphB2/ephrin-B1 signalling in the development and progression of obesity-associated colorectal cancer. Oncol Lett. (2022) 24:316. doi: 10.3892/ol.2022.13436 35949596 PMC9353875

[143] Jang BG Kim HS Bae JM Kim WH Hyun CL Kang GH. Expression profile and prognostic significance of EPHB3 in colorectal cancer. Biomolecules. (2020) 10:602. doi: 10.3390/biom10040602 32294981 PMC7226026

[144] Rönsch K Jägle S Rose K Seidl M Baumgartner F Freihen V. SNAIL1 combines competitive displacement of ASCL2 and epigenetic mechanisms to rapidly silence the EPHB3 tumor suppressor in colorectal cancer. Mol Oncol. (2015) 9:335–54. doi: 10.1016/j.molonc.2014.08.016 PMC5528665 25277775

[145] Park SH Jo MJ Kim BR Jeong YA Na YJ Kim JL. Sonic hedgehog pathway activation is associated with cetuximab resistance and EPHB3 receptor induction in colorectal cancer. Theranostics. (2019) 9:2235–51. doi: 10.7150/thno.30678 PMC6531304 31149041

[146] Lv J Xia Q Wang J Shen Q Zhang J Zhou X. EphB4 promotes the proliferation, invasion, and angiogenesis of human colorectal cancer. Exp Mol Pathol. (2016) 100:402–8. doi: 10.1016/j.yexmp.2016.03.011 27072105

[147] Xu C Gu L Kuerbanjiang M Jiang C Hu L Liu Y. Adaptive activation of EFNB2/EPHB4 axis promotes post-metastatic growth of colorectal cancer liver metastases by LDLR-mediated cholesterol uptake. Oncogene. (2023) 42:99–112. doi: 10.1038/s41388-022-02519-z 36376513 PMC9816060

[148] Krasnoperov V Kumar SR Ley E Li X Scehnet J Liu R. Novel ephB4 monoclonal antibodies modulate angiogenesis and inhibit tumor growth. Am J Pathol. (2010) 176:2029–38. doi: 10.2353/ajpath.2010.090755 PMC2843490 20133814

[149] Kadife E Ware TMB Luwor RB Chan STF Nurgali K Senior PV. Effects of EphB4 receptor expression on colorectal cancer cells, tumor growth, vascularization and composition. Acta Oncol (Madr). (2018) 57:1043–56. doi: 10.1080/0284186X.2018.1429650 29368976

[150] Ferrara N Hillan KJ Gerber H-P Novotny W. Discovery and development of bevacizumab, an anti-VEGF antibody for treating cancer. Nat Rev Drug Discovery. (2004) 3:391–400. doi: 10.1038/nrd1381 15136787

[151] Guijarro-Muñoz I Sánchez A Martínez-Martínez E García JM Salas C Provencio M. Gene expression profiling identifies EPHB4 as a potential predictive biomarker in colorectal cancer patients treated with bevacizumab. Med Oncol. (2013) 30:572. doi: 10.1007/s12032-013-0572-1 23579861

[152] Gurniak CB Berg LJ. A new member of the Eph family of receptors that lacks protein tyrosine kinase activity. Oncogene. (1996) 13(4):777–86. 8761299

[153] Xiao T Xiao Y Wang W Tang YY Xiao Z Su M. Targeting ephA2 in cancer. J Hematol Oncol. (2020) 13:114. doi: 10.1186/s13045-020-00944-9 32811512 PMC7433191

[154] Gan HK Parakh S Lee FT Tebbutt NC Ameratunga M Lee ST. A phase 1 safety and bioimaging trial of antibody DS-8895a against EphA2 in patients with advanced or metastatic EphA2 positive cancers. Invest New Drugs. (2022) 40:747–55. doi: 10.1007/s10637-022-01237-3 35404015

